# Pharmacological Treatment of Chemotherapy-Induced Neuropathic Pain: PPARγ Agonists as a Promising Tool

**DOI:** 10.3389/fnins.2019.00907

**Published:** 2019-08-28

**Authors:** Nara Lins Meira Quintão, José Roberto Santin, Luis Carlos Stoeberl, Thiago Patrício Corrêa, Jéssica Melato, Robson Costa

**Affiliations:** ^1^School of Heath Science, Universidade do Vale do Itajaí, Itajaí, Brazil; ^2^School of Pharmacy, Federal University of Rio de Janeiro, Rio de Janeiro, Brazil; ^3^Wolfson Centre for Age-Related Diseases, King’s College London, London, United Kingdom

**Keywords:** chemotherapy, platinum, taxane, nuclear receptor, neuropathy, chronic pain, side effects, quality of life

## Abstract

Chemotherapy-induced neuropathic pain (CINP) is one of the most severe side effects of anticancer agents, such as platinum- and taxanes-derived drugs (oxaliplatin, cisplatin, carboplatin and paclitaxel). CINP may even be a factor of interruption of treatment and consequently increasing the risk of death. Besides that, it is important to take into consideration that the incidence of cancer is increasing worldwide, including colorectal, gastric, lung, cervical, ovary and breast cancers, all treated with the aforementioned drugs, justifying the concern of the medical community about the patient’s quality of life. Several physiopathological mechanisms have already been described for CINP, such as changes in axonal transport, mitochondrial damage, increased ion channel activity and inflammation in the central nervous system (CNS). Another less frequent event that may occur after chemotherapy, particularly under oxaliplatin treatment, is the central neurotoxicity leading to disorders such as mental confusion, catatonia, hyporeflexia, etc. To date, no pharmacological therapy has shown satisfactory effect in these cases. In this scenario, duloxetine is the only drug currently in clinical use. Peroxisome proliferator-activated receptors (PPARs) belong to the class of nuclear receptors and are present in several tissues, mainly participating in lipid and glucose metabolism and inflammatory response. There are three PPAR isoforms: α, β/δ and γ. PPARγ, the protagonist of this review, is expressed in adipose tissue, large intestine, spleen and neutrophils. This subtype also plays important role in energy balance, lipid biosynthesis and adipogenesis. The effects of PPARγ agonists, known for their positive activity on type II diabetes mellitus, have been explored and present promising effects in the control of neuropathic pain, including CINP, and also cancer. This review focuses largely on the mechanisms involved in chemotherapy-induced neuropathy and the effects of the activation of PPARγ to treat CINP. It is the aim of this review to help understanding and developing novel CINP therapeutic strategies integrating PPARγ signalling.

## Introduction

Cancer is in the second position in the ranking of death causes after heart diseases across the globe and despite the huge efforts to implement novel chemotherapy strategies, the disease remains one of the major concerns worldwide (Bray et al., 2018). In 2012, the global number of new cases of cancer was 14.1 million, and the corresponding number of deaths was 8.2 million (Torre et al., 2015). For the year of 2018, according to The International Agency for Research on Cancer (IARC), 18.1 million of new cancer cases were estimated, followed by 9.6 million of deaths (Bray et al., 2018). The growing incidence and mortality of cancer is a result of population growth and ageing, besides changes in reproductive factors and unhealthy habits associated with economic development and urbanisation (Ferlay et al., 2015).

Approximately one-half of the cancer cases and deaths occurred in Asia, followed by Europe (23.4% of the cases and 20.3% of the deaths) and Northern America (21% of the cases and 14.4% of the deaths) (Bray et al., 2018; Ferlay et al., 2019). Lung, prostate and colorectal cancer were the most commonly diagnosed types of cancer among men, and lung cancer is the responsible for the greater number of deaths. The most frequent types of cancer in women were breast, colorectal and lung cancer, being breast cancer the top of five in cause of death (Bray et al., 2018).

Although the improvement of cancer survival by the aggressive treatments, new anti-cancer drugs are also responsible for serious side-effects on daily life that can last for many years. Cancer survivors suffer more from functional impairment, involving reduced mobility, than individuals without cancer history. The functional declines associated with cancer are linked to limited survival (Winters-Stone et al., 2017). Neurotoxicity to the peripheral (PNS) nervous system is an emerging side effect of cancer chemotherapy with no existing effective treatments (Brown et al., 2019). Chemotherapy-induced peripheral neurotoxicity (or neuropathy) is the most dose-limiting side effect of anti-cancer drugs, such as paclitaxel, vincristine and oxaliplatin, drugs widely used for treating several tumours. Peripheral neuropathy usually manifests as painful symptoms, characterising a neuropathic pain syndrome. However, it can progress to loss of sensory perception in the most severe cases. Additionally, motor and/or autonomic peripheral neuropathy can also occur (Brown et al., 2019). Chemotherapy-induced neuropathic pain (CINP) severally impairs the patient’s quality of life and leads to dose reduction or even treatment cessation (Miltenburg and Boogerd, 2014).

Recent pre-clinical studies have shown the efficacy of activators of the peroxisome proliferator-activated receptor gamma (PPARγ), known as glitazones, on neuropathic pain models (Okine et al., 2019). Therefore, glitazones might become new and effective pharmacological approaches to prevent CINP. In the present review, we will address pathophysiological mechanisms of CINP, its current pharmacological treatment and the use of PPARγ activators as potential therapeutic tools to manage CINP.

## Chemotherapy

The novel insights into the biology of cancer have been translated into improvements in clinical care at fast pace over the past 15 to 20 years. The introduction of sophisticated molecular tools, which interrogate both cancer diseases and patients, has led to a steady stream of new therapeutic interventions and altered the natural history of several solid tumours and heamatopoietic malignancies (Doroshow and Kummar, 2014).

The causes of cancer include damage and/or mutations in the cells’ genetic material associated with environmental or inherited factors, leading to uncontrolled cell proliferation. For cancers with local and non-metastatic profile, surgery and radiotherapy are the primary treatments choice. However, anti-cancer drugs, mainly chemotherapy, are the choice for treating metastatic cancers, since they are able to diffuse through the body (Hanahan and Weinberg, 2011). Anticancer drugs are toxic for cancer cells and inhibit their fast proliferation; however, they are not selective and also inhibit the growth of normal cells, leading to undesirable side effects commonly observed in cancer treatment. Chemotherapy has progressed towards more effective treatments, including the combination of drugs and new approved anticancer drugs, such as platinum analogues, paclitaxel and other agents (Perez-Herrero and Fernandez-Medarde, 2015).

**FIGURE 1:**
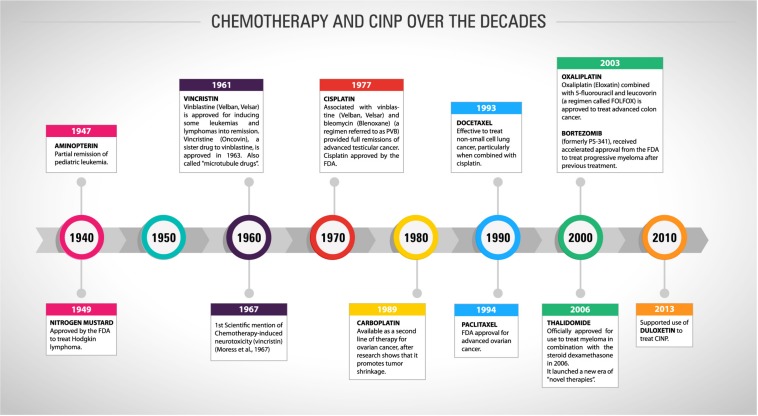
Timeline showing the history of chemotherapy and cancer treatment and the first mention of CINP and its recommended treatment over the last eight decades.

The development of chemotherapy drugs began in animal models in the twentieth century, but only during World War II the first reports of curative effects appeared. Advances in research and the recognition of oncology as a medical specialty allowed the creation of the first protocol to treat advanced cases of childhood leukaemia and Hodgkin’s disease in the 1960s and 1970s (Devita and Chu, 2008). Figure 1 shows the timeline of the FDA approval for chemotherapies over the last seven decades. The drug combination, using doxorubicin, bleomycin, vinblastine and dacarbazine, remains nowadays as the standard treatment for the management of Hodgkin’s lymphomas. At the same time, other drugs, such as methotrexate and cyclophosphamide, were included in the profile of cancer treatments (Chabner and Roberts, 2005). Chemotherapy included as adjunct to the surgical management of breast and colorectal tumours also began to spread in the same decade. With this new approach, the patient’s survival drastically increased (Bonadonna et al., 1976).

In the 1980s, the use of cisplatin (first-generation platinum) was started for the treatment of testicular cancer. After a good clinical response, its use was extended to ovarian, lung, head and neck and uterine cervix tumours (Carozzi et al., 2015). The main mechanism of action of cisplatin is the formation of a DNA-cisplatin adduct, which distorts the double helix of DNA and thus changes its structure. This effect induces cell death by apoptotic and necrotic processes (Jung and Lippard, 2007). Five years after the introduction of cisplatin, carboplatin, a second-generation platinum, emerged clinically. Carboplatin differs from cisplatin by the presence of a carboxylate-type binder in its chemical structure. The greater excretion through the urine, greater solubility in water and lower reactivity confer the carboplatin less toxicity when compared to the first-generation platinum (Wheate et al., 2010). Its efficacy in relation to cisplatin is seen mainly in cases of lung carcinomas (Pasetto et al., 2006). Oxaliplatin is the third-generation platinum which differs from cisplatin by the presence of an oxalate leaving group and a DACH (diaminocyclohexane) linker. Oxaliplatin is effective in cisplatin-resistant tumours because the DNA repair system does not recognise its adducts and is widely used in colorectal cancer (Pasetto et al., 2006). Neves and Vargas (2011) pointed to epidemiological data demonstrating a large scale of use of platinum (monotherapy or in combination with other drugs) in clinical oncology, ranging from 40 to 80% of the malignant tumours described cases.

Paclitaxel, the taxanes prototype, was firstly isolated in 1971 as part of a National Cancer Institute programme investigating a large range of plant extracts. It was initially isolated from *Taxus brevifolia* bark. Docetaxel, from the same class of anticancer drugs, was semi-synthetically obtained and presents higher solubility in water than paclitaxel. Taxanes are effective against breast, lung, ovarian, cervical and pancreatic cancers and Kaposi sarcoma (Weaver, 2014). The pharmacological effect of paclitaxel consists in its ligation to cytoplasm polymerised tubulins, interrupting G2 phase of cell cycle and, then, stabilising the microtubules. This interaction with tubulins also causes mitochondrial damage by opening the mitochondrial permeability transition pore, that has β-tubulin in its constitution, increasing the Ca^2+^ efflux and eventually apoptotic or necrosis cell death (Jordan and Wilson, 2004). This impairment does not occur exclusively in cancer cells, what reflect the numerous side effects experienced by the patients, including myelosuppression, hypersensitivity responses and, the most important, neuropathic pain (Carozzi et al., 2015).

With the best understanding of the genetic and phenotypic alterations of the tumours, the modalities of systemic treatments in oncology were expanded, being reinforced by the immunotherapy. It has been found that cancer cells in some specific types of cancer express on their surface proteins that could be used as targets for modulation and disruption of the tumour expansion process. Although cancer cells are highly genetically unstable, immunotherapy has been successfully used to manage numerous tumour types (Martin et al., 2015a). Toxicological assays that compared chemotherapy agents with immunomodulatory regimens in oncology concluded that the last has a greater safety in clinical applicability due to their well-defined targets, unlike chemotherapy agents that are less specific (Waldmann, 2003). In the current scenario, the greatest difficulty of immunotherapy is to adjust and handle enough monoclonal antibodies to reach the tumour site, so that its effect is potentiated. In addition, it is also required that the target of the monoclonal antibody should be highly specific and sufficiently expressed by the tumour cell, in addition of being directly involved with the cancer genesis (Guimaraes et al., 2008). Despite significant advances in cancer treatment with the discovery of immunotherapy, for some cancers, chemotherapy remains as gold standard treatment.

## Side Effects of Chemotherapy

Cytotoxic agents have narrow therapeutic indexes, with limited selectivity against cancer cells and high toxicity potential; consequently, anti-cancer drugs have limited efficacy at doses that are acceptable for most patients (Borcoman and Le Tourneau, 2016). Side effects of chemotherapy remain the major concern for both patients and clinicians despite the increase in efficacy and survival rates with the current treatments. The current approaches to counteract the side effects of chemotherapy are not completely effective, usually do not address long-term consequences or can induce other side effects (Nurgali et al., 2018).

Nausea and vomiting are the most dreaded side effects for patients who initiate anti-cancer chemotherapy. The current treatments to control acute chemotherapy-induced nausea and vomiting (CINV) are effective for most patients; however, the management of delayed CINV is more difficult to obtain (Andrews and Sanger, 2014). Mucositis is also an important side effect of anti-cancer drugs. Both oral and gastrointestinal mucositis can cause local ulceration and pain, leading to anorexia, malabsorption, weight loss, anaemia, fatigue and increased risk of sepsis. Despite many efforts of the scientific community, safe and effective treatments are still lacking to treat mucositis (Abalo et al., 2017). Other side effects of chemotherapeutic agents include hypersensitivity reactions to carboplatin in children with solid tumours; chronic subclinical skeletal muscle toxicity caused by oxaliplatin; and nephrotoxicity, ototoxicity and increased risk of cardiovascular disease in patients treated with cisplatin (Malik et al., 2016).

Central neurotoxicity induced by anticancer drugs can lead to persistent cognitive impairment, which has been associated with alterations in circulating factors and cerebrospinal fluid constituents, and occurrence of genetic polymorphisms. Additionally, peripheral neurotoxicity caused by many anti-cancer drugs, including platinum-based agents, vinca alkaloids and taxanes, can lead to neuropathic pain. These side effects can last many years after discontinuation of treatment and reduce the quality of life of cancer survivors. In addition, long term CINP is associated with depression, anxiety and insomnia. Therefore, the preventive and therapeutic strategies for CINP are an urgent need (Zhou et al., 2018).

## Chemotherapy-Induced Neuropathic Pain

Chemotherapy-induced neuropathic pain is essentially caused by injury to the somatosensory nervous system after anticancer drug treatment, and it is one of the major causes of neuropathic pain in clinical practice (Colvin, 2019). The incidence of CINP is variable among the studies with up to 81 and 98% for paclitaxel and oxaliplatin, respectively (Hershman et al., 2011; Gilchrist et al., 2017; Gebremedhn et al., 2018; Molassiotis et al., 2019). The occurrence of CINP may change according to number of cycles and duration of treatments, drug chemical structure, age, prescription of other neurotoxic drugs and presence of predisposing conditions such as alcoholism, diabetes or pre-existing neuropathy (Argyriou et al., 2014; Kerckhove et al., 2018). Sensory symptoms usually manifest as spontaneous or evoked abnormal sensations such as paraesthesia, dysesthesias, numbness, burning, shooting or electric shock sensations, as well as allodynia or hyperalgesia evoked by mechanical or thermal stimuli. The symptoms usually affect the extremities of the upper and lower limbs (“stocking and glove” distribution) and progress to the proximal regions of the body (Miltenburg and Boogerd, 2014; Colvin, 2019).

Chemotherapy-induced neuropathic pain can manifests initially as an acute pain syndrome, with sensory symptoms arising during or just after drug administration, and progress to a chronic neuropathy after repetitive treatment cycles. Regarding the duration of sensory symptoms, acute neuropathy generally subsides between treatments, while chronic neuropathy can persist for months or years (Colvin, 2019). Indeed, 47% of patients treated with anti-cancer drugs still experience peripheral neuropathy symptoms after 6 years of treatment termination (Winters-Stone et al., 2017). Chronic pain severely impairs the quality of life of cancer patients, reminding them of time they had cancer and that the disease may return (Binder and Baron, 2016). The available pharmacotherapies for CINP are poorly effective and associated with numerous side-effects. However, the search for more effective treatments is difficult as the physiopathology of CINP involves a complex machinery (for review see, Sisignano et al., 2014). Therefore, a deep knowledge of the molecular mechanisms involved in CINP is crucial to provide new molecular mechanism-based therapies instead of simply treating symptoms.

Several pathophysiological mechanisms have been described for CINP including mitochondrial dysfunction, changes in calcium homeostasis, oxidative stress, activation of apoptotic pathways, loss of myelinated and unmyelinated fibres, activation of the immune system and increased ion channel expression and activity. Comprehensive analysis of the pathophysiological mechanisms associated with CINP have already been performed elsewhere, and readers are invited to consult these reviews (for review see, Sisignano et al., 2014; Fukuda et al., 2017; Starobova and Vetter, 2017; Trecarichi and Flatters, 2019; Zajaczkowska et al., 2019). Despite some specific neurotoxic effects, anticancer drugs have important and mutual pathophysiological mechanisms that contribute to the development of CINP. Herein, we will present a possible sequence of events connecting the common mechanisms described for CINP associated with different anticancer drugs.

Peripheral sensory neurons are vulnerable to the toxic action of anti-cancer drugs as the PNS is devoid of a complex vascular-nerve barrier, allowing the diffusion of systemic-administered drugs to the dorsal root ganglia (DRG) (Abram et al., 2006; Sapunar et al., 2012). The damage to the cellular bodies of sensory neurons leads to the degeneration of myelinated fibres (mainly) and, consequently, inflammatory process, overactivity of remaining fibres and central sensitisation (Fukuda et al., 2017). In fact, axonopathy and loss of epidermal innervation were described after the treatment with paclitaxel, vincristine or ixabepilone (Lapointe et al., 2013). Furthermore, peripheral and central inflammatory responses have been described as important mechanisms of pain, including paclitaxel-, vincristine- and oxaliplatin-induced neuropathic pain (Marotta et al., 2009; Ji et al., 2013; Janes et al., 2015; Makker et al., 2017; Segat et al., 2017; Costa et al., 2018; Manjavachi et al., 2019). Additionally, these drugs increase the activity of both voltage-dependent calcium (Cav) and sodium (Nav) channels, and transient receptor potential (TRP) channels in peripheral nerves (Sisignano et al., 2014). Central neuronal sensitisation, marked by phosphorylation and activation of *N*-methyl-D-aspartate (NMDA) receptor, has also been described for CINP (Pascual et al., 2010; Mihara et al., 2011; Ji et al., 2013).

Regarding the cellular mechanisms of CINP, mitochondrial damage has been reported as a key component of the damage to sensory neurons in the DRG after the treatment with different anticancer drugs. It has been widely reported that paclitaxel, vincristine and oxaliplatin cause mitochondrial dysfunction and, consequently, increased production of reactive oxygen species (ROS) in the DRG (Duggett et al., 2016, 2017; Gong et al., 2016; Vashistha et al., 2017; Khasabova et al., 2019). Chemotherapy causes impairment in cellular respiration and decreases the production of adenosine triphosphate (ATP), and promoting mitochondrial respiration and restoring mitochondrial bioenergetics has protective effect on CINP (Bennett et al., 2014; Toyama et al., 2018). Additionally, the level and activity of superoxide dismutase (SOD) and catalase, two important antioxidant enzymes, are reduced by the treatment with anti-cancer drugs generating an imbalance between oxidant and antioxidant molecules (Janes et al., 2013; Duggett et al., 2016; Khasabova et al., 2019). Together, these effects trigger cellular apoptotic pathways that lead to the degeneration of peripheral sensory fibres and related inflammatory process (Areti et al., 2014; Fukuda et al., 2017).

Once oxidative stress is a key event in the physiopathology of CINP, antioxidant strategies are believed to be effective alternatives for preventing the development of CINP. Studies with animal models have been performed in order to determinate the effect of several antioxidant agents on CINP (Carvalho et al., 2017). Notably, calmangafodipir, an antioxidant and neuroprotective agent, has shown to prevent oxaliplatin-induced neuropathic pain in a double-blinded randomised phase II clinical trial (Glimelius et al., 2018).

## Treatment of CINP

**FIGURE 2:**
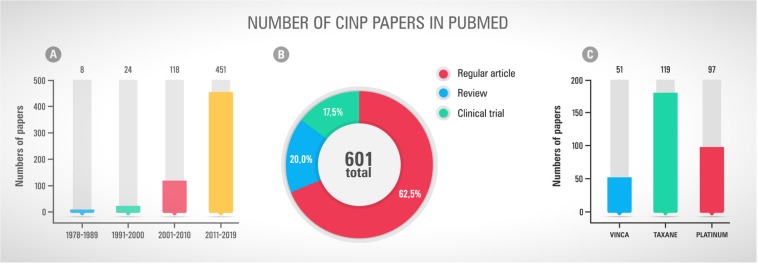
**(A)** Number of scientific papers tagged “chemotherapy” and “peripheral neuropathy” and “pain” and “cancer” in Pubmed by decade of publication. **(B)** Types of publications, including regular article, review and clinical trial, found using the terms mentions in the first graph. **(C)** Number of papers mentioning the chemotherapy classes Vinca, Taxane and Platinum. Accessed in May 26^th^, 2019.

As mentioned before, the neurotoxicity and chronic pain induced by chemotherapy treatments are important adverse effects that must be considered, once they could compromise the cancer treatment and the post-treatment patients’ quality of life. The first study mentioning neuropathic pain in cancer-patients dates from 1967 (Figure 1), where three case reports were presented with necropsy findings linking the neurological symptoms with vincristine neurotoxicity (Moress et al., 1967). Since then, as presented in Figure 2, the number of papers has grown year by year focussing on both the pathophysiological mechanisms of CINP and new treatments (Figure 2A). Most of publications involve regular articles (Figure 2B) and the great majority mention taxanes, followed by platinum drugs (Figure 2C).

Despite the growing search for new drugs, the American Society of Cancer and American Society of Clinical Oncology (ASCO), until now, do not endorse the prescription of other pharmacological therapy or nutraceutical besides duloxetine. The reason for that consensus is based on the absence of evidence for efficacy and safety for other therapies (Hershman et al., 2014; Hou et al., 2018). Table 1 reunites all clinical trials that investigated or plans to investigate pharmacological strategies to prevent or treat CINP. Several classes of drugs already known to be effective in the neuropathic pain control, such as antidepressants and anticonvulsants, have been pre-clinically and clinically tested and surprising the specialists with their absence of effect. We can cite gabapentin, pregabalin and amitriptyline (Table 1). This scenario leads to believe that it is a pathological condition with a profile significantly different from other neuropathies and, unfortunately, difficult to manage, since the great majority of the trials has failed to reduce the symptoms. New clinical trials are being conducted to evaluate new strategies focussing on the main mechanism of CINP, including oxidative stress, mitochondrial impairment and ion channels (more specifically TRP and Na^+^ channels) (Table 1).

**TABLE 1:** Clinical trials for CIPN treatment using drugs or nutraceuticals around the world.

**Study**	**Trial #**	**Country**	**Subjects**	**Start (yr)**	**Status**	**Remarks**	**References**
**Antidepressant**							
Duloxetin (Sinbalta^®^)	UMIN000017647	Japan	70	2015	Ongoing	Phase III	Matsuoka et al., 2017
	UMIN 000011554	Japan	34	2013	Completed	Phase II – pilot randomised trial; reduction of pain symptoms	Hirayama et al., 2015
	NCT00489411	United States	231	2008	Completed	Phase III – significant reduction of pain score	Smith et al., 2013
	NCT00489411	United States	106	2008	Completed	Phase III – significant reduction of pain score	Smith et al., 2017
Amitriptyline	–	Finland	114	2003	Completed	Preventive protocol; use not supported	Kautio et al., 2008
	–	Finland	44	2002	Completed	Therapeutic protocol; improve symptoms of CINP	Kautio et al., 2008
**Anticonvulsant**							
Gabapentin (Neurontin^®^)	NCT00027963	United States	100	2002	Completed	Phase III – use not supported	Rao et al., 2007
Pregabalin (Lyrica^®^)	NCT02394951	United States	26	2015	Completed	Results not mentioned	
	–	United States	46	2012	Completed	Pilot study; PTX-treated patients; use not supported	Shinde et al., 2016
	NCT00380874	Europe/Asia^a^	61	2006	Terminated	Phase IV	
	NCT00407511	Latin America^b^	121	2006	Completed	Phase IV – not conclusive for CINP	Xochilcal-Morales et al., 2010
Lamotrigine (Lamictal^®^)	–	United States	131	2004	Completed	Use not supported	Rao et al., 2008
Ethosuximide (Zarontin^®^)	NCT01278004	United Kingdom	15	2011	Completed	Phase II – results not mentioned	
	NCT02100046	France	114	2014	Completed	Phase II – use not supported	Kerckhove et al., 2018
**Antipsychotic**							
Loxapine	NCT02820519	United States	4	2016	Terminated	Phase II – intolerable high amount of adverse effects	
**Associations**							
Memantine XR-pregabalin combination	NCT03272919	United States	20	2017	Recruiting	Observational study	
Baclofen-Amitriptyline Hydrochloride-Ketamine gel (BAK)	NCT00516503	United States	208	2007	Completed	Phase III – reduced pain symptom	Barton et al., 2011
**Opioid**							
Dextromethorphan (Robitussin^®^)	NCT02271893	France	40	2014	Recruiting	Phase II	Martin et al., 2015b
**Neuroprotector**							
Olesoxime (TRO19622)	NCT00876538	France	17	2009	Completed	Phase II – results not mentioned	
Calmangafodipir (PledOx^®^)	NCT03654729	United States	420	2018	Recruiting	Phase III	
	NCT01619423	United States	186	2012	Completed	Phase I and II-OXA-treated patients; reduced pain symptom	Glimelius et al., 2018
Leteprinim (Neotrofin^®^)	NCT00041795	United States	50	2002	Completed	Phase II – results not mentioned	
Glutathione		United States	195	2009	Completed	Phase III – PTX-treated patients; use not supported	Leal et al., 2014
**Weight loss**							
Lorcaserin (Belviq^®^)	NCT03812523	United States	50	2019	Not yet recruiting	Phase II – OXA-treated patients	
**Cannabinoid agonists**							
Cannabinoids	NCT03782402	United States	100	2019	Not yet recruiting	Phase II – taxane-induced neuropathy	
Nabiximol (Sativex^®^)	NCT00872144	Canada	16	2009	Completed	Phase III – reduced pain symptoms	Lynch et al., 2014
Nabilone	NCT00380965	United States	23	2006	Completed	Phase IV – results not mentioned	
**Toxins**							
Botulinum Toxin A	NCT03571334	United States	40	2018	Not yet recruiting	Phase II	
Tetrodotoxin	NCT01655823	United States	125	2012	Terminated	Phase II – interim analysis determined the procedure to phase III trial	
**Anaesthetic**							
Lidocaine	NCT03254394	United States	38	2017	Recruiting	Phase I/II – OXA-treated patients	
**TRPs agonist**							
Capsaicin 8% patch (Qutenza^®^)	NCT03317613	France	84	2017	Recruiting	Phase II	
	–	Poland	18	2013	Completed	OXA-treated patients; reduction of pain symptoms	Filipczak-Bryniarska et al., 2017
Menthol	NCT01855607	United States	60	2013	Unknown	Phase II	
**Nutraceutic**							
L-Carnitine L-tartrate	NCT00754767	United States	2	2007	Terminated	Phase IV – unable to accrue study participants	
Acetyl L-carnitine	NCT01526564	China	239	2012	Completed	Phase III – results not mentioned	
	NCT00775645	United States	437	2008	Completed	Phase III – use not supported	Hershman et al., 2013
	NCT0058191	United States	32	2004	Completed	Phase II – use not supported	Callander et al., 2014
Nicotinamide Riboside	NCT03642990	United States	39	2019	Recruiting	Phase II	
Omega-3/Vitamin D3	NCT02294149	Canada	600	2014	Unknown	Phase III	
Vitamin E	NCT00363129	United States	207	2006	Completed	Phase III – use not supported	Kottschade et al., 2011
α-Lipoic acid	–	United States	462		Completed	Use not supported	Guo et al., 2014
**Antibiotic**							
Minocycline hydrochloride	NCT02297412	United States	47	2014	Completed	Phase II – PTX-treated patients; use not supported	Pachman et al., 2017

*PTX, paclitaxel; OXA, oxaliplatin; ^*a*^Pfizer (Australia, Germany, Italy, Spain, Korea, Taiwan); ^*b*^Pfizer (Colombia, Equator, Mexico, Peru, Venezuela). Source: Pubmed and www.ClinicalTrials.gov, accessed in April 26^th^ 2019.*

**FIGURE 3:**
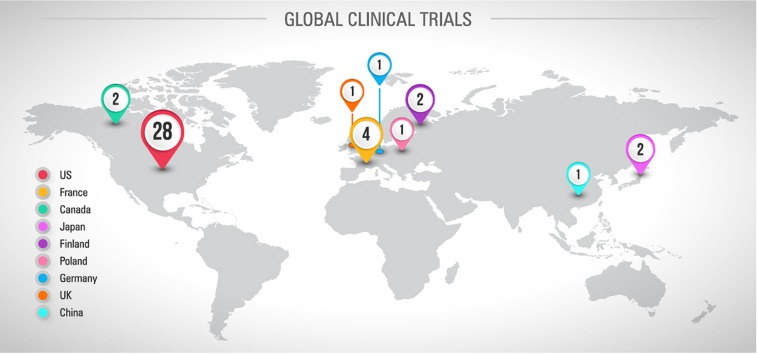
Global distribution of registered and published clinical trials involving CINP and drugs or nutraceuticals. Sources: Pubmed and www.ClinicalTrials.gov, accessed in April 26^th^, 2019.

**FIGURE 4:**
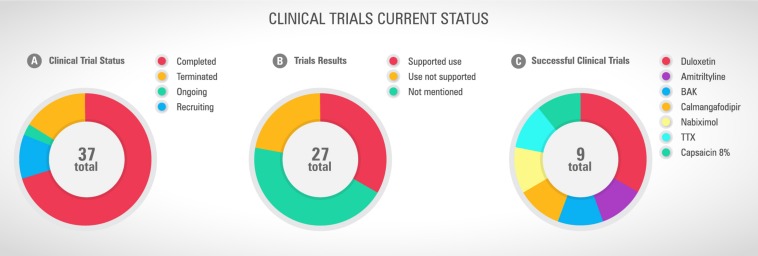
**(A)** Current clinical trial status, **(B)** studies successfully completed and their results, and **(C)** drugs or nutraceuticals with recommended use. Sources: Pubmed and www.ClinicalTrials.gov, accessed in April 26^th^, 2019.

Looking at the current scenario resumed in the Figure 3, there are 42 registered clinical trials investigating new pharmacological strategies to treat or prevent CINP. Most of them (*n* = 28) are conducted in the US. Only 27 studies have been completed and 4 have been terminated due to different reasons, including absence of participants and important side effects (Figure 4A). By the total, 9 studies support the therapy use against 12 that do not support it (Figure 4B). One third of the studies with successful results supports the use of duloxetine (Figure 4C). Observing this data, it is clear why the only therapy indicated by ASCO to treat CINP is duloxetine, all based on evidence of efficacy and safety.

## Peroxisome Proliferator-Activated Receptors

Peroxisome proliferator-activated receptors (PPARs) are important members of the nuclear receptor family that cause the activation of several genes by acting as ligand-activated transcription factor (Berger and Moller, 2002). In mammals, there are three different PPAR isoforms: alpha (α), beta/delta (β/δ), and gamma (γ), which are differentially expressed in several tissues (Heneka and Landreth, 2007). PPARα (encoded by NR1C1) is ubiquitously expressed, but it is mostly found in tissues that present fatty acids high catabolic amounts, such as adipose tissue and liver, among others. It is also expressed in the lung, placenta, intestine, pancreas and skeletal muscle. Furthermore, PPARβ/δ (encoded by NR1C2) is also ubiquitously expressed and low levels are found in several tissues, such as muscle, adipose tissue and liver. PPARγ (encoded by NR1C3) has three different isoforms (γ1, γ2, and γ3) that display differences in tissue expression for each isoform: γ1 has ubiquitous tissue expression, γ2 is mostly expressed in adipose tissue, and γ3 is expressed mainly in colon, macrophages and adipose tissue (Han et al., 2017). Additionally, low levels of PPARγ were found in vascular smooth muscle, endothelium, hepatic stellate cells, bone marrow and neoplastic epithelial cells in breast, prostate, colon and bladder. This pattern of expression suggests that PPARγ may participate of many physiological and pathophysiological processes in different tissues (Guan and Breyer, 2001).

PPARs were originally identified in 1990 with the first cloning (PPARα) happening during molecular targeting for peroxisome proliferating agents in rodents (Issemann and Green, 1990). Since then, several fatty acids and by-products, including eicosanoids, have been identified as PPARs ligands and have also been shown to target many synthetic compounds currently used to treat diabetes and dyslipidaemias, such as thiazolidineodiones (TZDs), including pioglitazone and rosiglitazone, and fibrates (clofibrate) (Guan and Breyer, 2001; Seiri et al., 2019). Therefore, the knowledge of the molecular structure and physiological effects of these receptors becomes particularly important, both in the development and in the use of drugs to treat of metabolic diseases and others illness.

**FIGURE 5:**
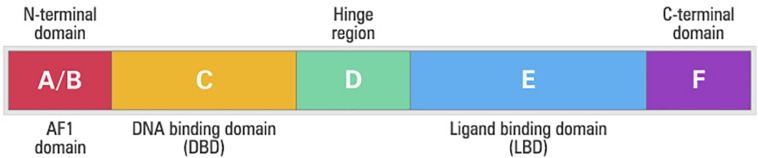
Structural and functional domains of the human peroxisome proliferator-activated receptors (PPARs). A/B, N-terminal A/B domain containing a ligand-independent activation function (AF1); C, DNA-binding domain (DBD); D, hinge region; E, ligand-binding domain (LBD) containing the ligand-dependent activation function, and F, C-terminal domain.

Independent of the PPAR type, all isoforms have similar structure (Korbecki et al., 2019). PPARs are composed of five different domains: A/B domain (amino-terminal region), domain C (DNA-DBD binding), domain D (hinge region), domain E (interaction with the linker – LDB) and domain F (Itoh et al., 2008; Figure 5). The amino-terminal (A/B) domain is extremely variable between the members of the nuclear receptor superfamily, both in size and amino acid sequence, and exhibits a transcriptional activation function that operates independently of the linker, termed activation function 1 (AF1) (Shao et al., 1998; Blanquart et al., 2003). The AF1 domain has an important role in the regulation of PPAR activity trough phosphorylation (Shao et al., 1998).

The domain C is the best-conserved part of the protein among the nuclear receptor family and its main function is the binding to DNA. This domain is in the central portion of the receptors and consists of two structural segments known as zinc fingers with nine cysteines. This domain controls gene expression through specific binding to the nucleotides sequences called the peroxisome proliferator responsive element (PPRE) after forming heterodimer with the retinoic X receptor (RXR) nuclear receptor (Guan and Breyer, 2001). There is a small region in the domain D that connects the DBD to the ligand-binding domain (LBD), or E domain, which is known as the hinge, allowing the rotation of the DBD in relation to the LBD (Guan and Breyer, 2001). The LDB domain is in the carboxy-terminal region and has several functions such as ligand recognition and homo and heterodimerisation of the receptor (Seiri et al., 2019). In addition to these functions, LBD contains a surface that is critical for transcriptional activation. After the activation of this region, called activation function 2 (AF-2), interaction with the co-activators occurs, which will allow the formation of the protein complex involved in the activation of the transcription (Guan and Breyer, 2001; Seiri et al., 2019).

Data from literature have shown that the transcription induced by PPARs is modulated by post-translational events, including phosphorylation, SUMOylation, ubiquitination and nitration (Van Beekum et al., 2009). However, the phosphorylation receives more attention by the researcher community for being the main determinant of PPARγ transcriptional activity, as already observed for oestrogen, progesterone and RXR receptors. However, its activity is determined by the intracellular localisation of the receptor, where nuclear migration leads to genomic effects, while cytosolic or cell membrane activation promotes DNA-independent effects (Cantini et al., 2010; Luconi et al., 2010). In fact, PPARγ could regulate the different metabolic situations, such as lipid and glycidic homeostasis, inflammation and also cell proliferation by specifically modulating genes expression. The genomic mechanism is based on gene transcription regulation, where a PPAR ligand-bound receptor interacts with the RXR on specific PPRE in the promoter of specific target genes and recruits co-activator complexes that modify chromatin structure, enabling assembly of transcriptional machinery on the promoter (Korbecki et al., 2019). However, some effects of PPARs are correlated with trans-repression mechanism, mainly the anti-inflammatory effects, blocking the transcription factors activity, such as NF-kB and AP-1 (Daynes and Jones, 2002; Pawlak et al., 2012). Known as non-genomic pathway, this effect of PPARs has been in the spotlight, where the mechanism did not involve enhancement or inhibition of gene transcription. The non-genomic effects are correlated to the fast modulation of intracellular activity, including kinases and phosphatases. Therefore, the mechanism that mediates rapid action is still controversial (Brown, 1981).

Independent of the mechanism, it is important to emphasise that PPAR, mainly PPARγ, is highly expressed in different types of cells. In the CNS, PPARγ have been described to be expressed in the cortex and spinal cord, and also in the microglia and astrocytes (Kainu et al., 1994; Cullingford et al., 1998; Cristiano et al., 2001; Benani et al., 2003; Bernardo et al., 2003; Moreno et al., 2004; for review see, Okine et al., 2019). However, PPARγ is more expressed in neurons than in astrocytes or microglia (Warden et al., 2016).

The PPARγ is the most well-studied member of the PPAR family of nuclear receptors, and both ligand-dependent and ligand-independent modes of modulation of its activity have been established. In this context, PPARγ modulation has been currently focussed in the market and in the scientific research of new drug discovery. The research is based mainly in metabolic and neurodegenerative disorders, and other conditions where CNS is affected as neuropathic pain (Okine et al., 2019).

## PPARγ Agonists to Treat Cinp

Thiazolidineodiones, or simply “glitazones,” belong to a class of compounds that activates PPARγ and can be employed to treat type 2 diabetes and metabolic syndrome (Sauer, 2015). Ciglitazone was the first drug described as an insulin sensitiser, and TZDs were recognised as PPARγ agonists in 1995. Two years later, the FDA approved the clinical use of troglitazone. In 1999, two new drugs, rosiglitazone and pioglitazone, entered in the hall of anti-diabetic drugs. Unfortunately, what seemed to be a future of success, ended with the troglitazone removed from the market in 2000 because of significant liver toxicity. On the other hand, pioglitazone and rosiglitazone remain in clinical practice, despite their association with increased bladder cancer risk and cardiovascular disease, respectively (Sauer, 2015).

In addition to the treatment of diabetes, PPARγ agonists have been considered potential therapeutic drugs to treat a large amount of neurological conditions, such as neurodegenerative diseases, traumatic injury, demyelinating diseases and chronic pain (Jin et al., 2013; Swanson et al., 2013; Vallee and Lecarpentier, 2016; Patel et al., 2017; Villapol, 2018). Recently, it was published a comprehensive review compiling all studies of PPAR agonists in different types of pain models (Okine et al., 2019). Pioglitazone, rosiglitazone and 15d-PGJ_2_ have been largely employed in pre-clinical studies using different models of neuropathic pain in rodents, showing anti-nociceptive effect by reducing oxidative stress and inflammation in the DRG and spinal cord (Table 2; Okine et al., 2019).

**TABLE 2:** Pre-clinical studies investigating PPARγ agonists effects in experimental neuropathic pain models.

**Glitazones**	**Model**	**Specie**	**Main effects**	**Mechanism of action**	**References**
Pioglitazone and Rosiglitazone	Spinal cord injury	Rats	Improvement of motor function recovery and prevention of heat hypersensitivity.	Reduction of neuronal damage, inflammation and myelin loss in the spinal cord.	Park et al., 2007
Pioglitazone	Partial sciatic nerve ligation	Mice	Reversal of mechanical allodynia and heat hyperalgesia.	Reduction of inflammation in the sciatic nerve, DRG and spinal cord.	Maeda et al., 2008
Rosiglitazone	Spared Nerve Injury	Rats	Reversal of mechanical and cold allodynia.	Transcription-independent mechanism in the spinal cord.	Churi et al., 2008
Rosiglitazone	Tibial and sural nerve transection	Rats	Attenuation of mechanical and cold hyperalgesia.	Inhibition of oxidative stress and inflammation in the sciatic nerve.	Jain et al., 2009
Rosiglitazone	Partial sciatic nerve ligation	Mice	Attenuation of mechanical allodynia	Regulation of macrophage infiltration and pro-inflammatory molecules production in the sciatic nerve.	Takahashi et al., 2011
Pioglitazone	Spared nerve injury	Rats	Prevention of mechanical and cold hypersensitivities	Inhibition of microglia and/or astrocyte activation in the spinal cord.	Morgenweck et al., 2013
Pioglitazone	Spinal nerve transection	Rats	Prevention of mechanical hypersensitivity	Inhibition of neuro-inflammation in spinal cord.	Jia et al., 2013
Rosiglitazone	Oxaliplatin-induced neuropathic pain	Rats	Prevention of mechanical and cold hyperalgesia	Prevention of oxidative stress in the DRG and spinal cord by increasing catalase activity.	Zanardelli et al., 2014
Pioglitazone	Spared Nerve Injury	Rats	Reversal of mechanical and cold allodynia	Inhibition of astrocyte activation by non-genomic mechanisms.	Griggs et al., 2015
Pioglitazone	Spinal nerve ligation	Rats	Prevention of mechanical, cold and heat hypersensitivities	Inhibition of oxidative stress, inflammation and apoptosis in the spinal cord.	Pottabathini et al., 2016
Pioglitazone	Trigeminal inflammatory compression	Mice	Attenuation of mechanical allodynia	Activation of PPARγ in the trigeminal brainstem sensory nucleus.	Lyons et al., 2017
Pioglitazone	Cisplatin-induced neuropathic pain	Mice	Reduction of mechanical and cold hyperalgesia	Reduction of oxidative stress in the DRG by increasing SOD activity.	Khasabova et al., 2019

The expression of PPARγ at both mRNA and protein levels was found in the spinal cord of rats, and the intrathecal injection of PPARγ agonists (15d-PGJ_2_ or rosiglitazone) was able to reverse mechanical allodynia induced by spare nerve injury (SNI) in rats, indicating that PPARγ is functionally expressed in the spinal cord (Churi et al., 2008). The immunoreactivity for PPARγ was also found in the mouse DRG and spinal cord neurons, as well as in sciatic nerve adipocytes, where the receptor was believed to mediate the anti-allodynic effect of pioglitazone by controlling inflammation (Maeda et al., 2008). Despite no data about the spinal levels of PPARγ in injured animals, a further study showed that PPARγ activity was not altered by SNI in rats, but it was significantly increased by the treatment of animals with R-flurbiprofen; the increase in PPARγ activity was proposed to be one of the mechanisms involved in the antinociceptive effect of R-flurbiprofen in the SNI model (Bishay et al., 2010). PPARγ was also suggested to meditate the antinociceptive effect of palmitoylethanolamide on the chronic constriction injury (CCI) model of neuropathic pain (Costa et al., 2008).

As previously mentioned, mitochondrial dysfunction, oxidative stress and, consequently, neuronal injury in the DRG and spinal cord are key events in the physiopathology of CINP. Therefore, TZDs could have beneficial effects on CINP by limiting some, if not all, of these events. In fact, several studies have proposed that the main mechanisms of action of PPARγ agonists are the protection of mitochondrial function and antioxidant activity, including the upregulation of mitochondrial oxidative phosphorylation and biogenesis, and improvement of endogenous oxidant defences (for review see, Corona and Duchen, 2016). Indeed, TZDs were able to protect cortical astrocytes and neuroblastoma derived cell line by promoting mitochondrial biogenesis (Dello Russo et al., 2003; Miglio et al., 2009). Also, ciglitazone reduced the oxidative stress in hippocampal neurons and, consequently, prevented the mitochondrial damage (Zolezzi et al., 2013). The protective effects of TZDs were also attributed to their ability of reducing apoptosis associated with oxidative stress (Hunter et al., 2007; Wang et al., 2011). In a rat model of spinal nerve ligation (SNL), pioglitazone alone or in association with ceftriaxone was able to ameliorate neuropathic pain by restoring the activity of mitochondrial enzyme complex activities, increasing the levels of reduced glutathione (GSH), superoxide dismutase (SOD) and catalase, and reducing oxidative damage in the rat spinal cord (Pottabathini et al., 2016). The treatment of rodents with oxaliplatin or cisplatin caused an imbalance between the oxidative stress and the level of antioxidant enzymes in the DRG and spinal cord of treated animals (Zanardelli et al., 2014; Khasabova et al., 2019). In these studies, rosiglitazone was effective in preventing oxaliplatin-induced mechanical and cold hyperalgesia by inhibiting oxidative stress and increasing catalase activity in the DRG and spinal cord of rats (Zanardelli et al., 2014). Additionally, in a recent publication Khasabova et al. (2019) demonstrated that pioglitazone reduced cisplatin-induced neuropathic pain in mice, suggesting the improvement of antioxidant enzymes activity and protection against oxidative stress as the main mechanisms. Besides, pioglitazone was able to increase the sensitivity of cancer cells to the chemotherapy, reducing the levels of its concentration to block cell proliferation (Khasabova et al., 2019). Taken together, these studies suggest that PPARγ agonists could prevent CINP and improve the efficacy of cancer chemotherapy.

Neuroinflammation in the spinal cord is an important imprint of neuropathic pain that contributes to the chronicity of pain. Studies using the mouse model of paclitaxel-induced neuropathic pain have shown increased immunostaining for Iba-1 (microglia marker) and augmented levels of NF-κB, cytokines and chemokines in the spinal cord of paclitaxel-treated mice (Segat et al., 2017; Manjavachi et al., 2019). Also, the release of kinins and the activation of their receptors (B_1_ and B_2_ receptors) in the spinal cord seem to be important for paclitaxel-induced neuropathic pain in mice (Costa et al., 2011). The blockage of spinal cord neuroinflammation using natural compounds, monoclonal antibodies or antagonists (for kinin B1, B2 or CXCR2 receptors) has been shown to prevent and revert pain-like behaviours in paclitaxel-treated mice (Costa et al., 2011, 2018; Segat et al., 2017; Manjavachi et al., 2019). Therefore, PPARγ agonists could be effective pharmacological tools to treat CINP by reducing the inflammatory process in the spinal cord or even in the DRG. In fact, the non-genomic activity of PPARγ has been extensively co-related with its anti-inflammatory property, characterising TZDs as important blockers of protein transcription (Sauer, 2015). Additionally, several studies have demonstrated an important link between the efficacy of PPARγ agonists on neuropathic pain and the suppression of inflammatory gene expression (including cytokines and cytokines) (Maeda and Kishioka, 2009; Freitag and Miller, 2014).

While DRGs and spinal cord are considered the most likely structures involved in the anti-nociceptive effect of TZDs, the cell types mediating their actions are not well-characterised. However, the expression of PPARγ in both neuronal and non-neuronal cells has been shown (Lu et al., 2011). PPARγ expressed in astrocytes was proposed to regulate oxidative stress, as the impairment in its activity reduced catalase activity, a key antioxidant defence enzyme (Di Cesare Mannelli et al., 2014). In the CNS, PPARγ activation reduced JNK and NF-KB signalling, as well as JAK/STAT pathway, modulating the activity of adaptive immune cells, myeloid cells and astrocytes (Daynes and Jones, 2002; Bright et al., 2008). PPARγ activation is also able to reduce the levels of inflammatory and neurotoxic mediators produced by macrophages and astrocytes and stimulate the infiltration of regulatory T cells (Ferret-Sena et al., 2018).

## Potential Effect of PPARγ Agonists on Cancer

In 2008, PPAR Research Journal published several reviews regarding the role of PPAR and its agonists in cancer and the possible mechanisms involving on it. Taking the last decade, approximately 1,400 regular articles and clinical trials have been published evaluating the involvement of PPARγ on cancer development and its modulation or even anti-cancer profile of PPARγ agonists (data extracted from PUBMED on 24^th^ July using the terms “PPARγ and cancer”). Considering the current year (2019), 14 studies revels the strong potential of PPARγ as target to promote reduction in neoplastic cell proliferation and migration. It demonstrates that this receptor and its signalling pathways are in the pipeline of new drugs for the treatment of patients with different types of cancer.

As previously described, pioglitazone was linked to a high risk of developing bladder cancer, which was seriously considered by the medical community in case of PPARγ prescription to patients with cancer or familiar history. Recently, Rochel et al. (2019) have shown that mutations in the PPARγ protein are responsible for the pro-oncogenic activity of the heterodimer PPARγ/RXRα, leading to bladder luminal cancer. This discovery reintroduces the receptor among the promisor new targets to treat cancer, and considering that co-morbidities, such as metabolic syndrome, have strong implication in cancer development, it becomes more significant.

Besides that, PPARγ gain more pros than cons with the studies that demonstrate the activity of their ligands as cancer suppressors. Here we are going to mention the important results obtained with PPARγ activation published only this year. Piccinin et al. (2019) demonstrated that the administration of PGC1, a PPARγ activator, was able to reduce the progression of hepatocellular carcinoma. The enhanced invasion and migration of colorectal cancer cells promoted by the microRNA-11 was reverted by the increase of PPARγ expression induced by Fatty Acid Binding Protein 4 (FABP4) activation (Zhao et al., 2019). In fact, it was previously shown that the inhibition of the oncogenic Src culminated in the enhancement of the axis FABP4/PPARγ, working as tumour repressor (Hua et al., 2019).

TZD18, a dual PPARγ/α ligand, reduced the growth and increased the apoptosis of human gastric cancer cells by increasing the expression of BAX and p27kip1 and decreasing Bcl-2 (Ma et al., 2019). Similar activity was observed for renal carcinoma cells (Wu et al., 2019), cutaneous squamous cell carcinoma cells (Wolff et al., 2019), non-small cell lung carcinoma (Liu and Fang, 1983; Ciaramella et al., 2019) and prostate cancer cells (Masure et al., 1983). Ciaramella et al. (2019) also correlated the anti-cancer activity of PPARγ to its effects on cancer microenvironment bioenergetics and metabolism.

Furthermore, the PPARγ was also implicated in the enhancement of doxorubicin cytotoxic effect of K562 resistant cells after treatment with ciglitazone, emphasising its important role in the multidrug resistance (MDR) activity. Additionally, Lv et al. (2019) demonstrated that PPARγ expression in cancer cells is related to favourable prognostic of patients with bladder cancer and that the *in vitro* and *in vivo* administration of pioglitazone or rosiglitazone was responsible for enhancing the cell cycle G2 arrest and apoptosis, followed by reduction in cell proliferation and tumour growth through PI3K-AKT pathway.

## Concluding Remarks

Safe and effective therapies to prevent or treat CINP are still an unmet clinical need. Drugs normally effective against chronic pain conditions, such as gabapentin and tricyclic antidepressants, failed to relieve CINP. The physiopathology of CINP involves a complex machinery where mitochondrial impairment and oxidative stress are key elements, leading to cell death, neuronal damage and inflammatory process. PPARγ agonists can protect cells against mitochondrial damage and the deleterious effect of oxidative stress, and interfere with the synthesis of important chemical mediators, such as cytokines and chemokines. Therefore, the use of PPARγ agonists to treat CINP have provoked the interest of scientists and clinicians. In fact, rosiglitazone and pioglitazone have shown antinociceptive effect on chronic pain models, including neuropathic pain induced by platinum-based drugs, by increasing the antioxidant defences and reducing oxidative stress. Additionally, PPARγ agonists have been pointed as potential pharmacological tools to suppress cancer progression. Therefore, the use of TZDs in the treatment of CINP could also have a positive impact on cancer treatment, what is favourable to the use of these drugs in cancer patients.

Despite being a promising pharmacological strategy, further studies are essential to support the use of TZDs in treatment of CINP. First, the effect of TZDs on neuropathic pain induced by other anticancer agents, such as paclitaxel or bortezomib, should also be addressed. Second, the mechanisms of action of these drugs on CINP must be fully characterised (for example, the effect of TZDs on neuroinflammation associated with CINP has not yet been evaluated). Finally, joint effort of chemists, pharmacologists and physicians should prioritise the search for new PPARγ agonists, with reduced side effects, good permeability at blood brain barrier and positive effects in reducing CINP.

## Author Contributions

All authors listed have made a substantial, direct and intellectual contribution to the work, and approved it for publication.

## Conflict of Interest Statement

The authors declare that the research was conducted in the absence of any commercial or financial relationships that could be construed as a potential conflict of interest.
